# Serum Levels of Soluble Urokinase Plasminogen Activator Receptor Predict Tumor Response and Outcome to Immune Checkpoint Inhibitor Therapy

**DOI:** 10.3389/fonc.2021.646883

**Published:** 2021-04-01

**Authors:** Sven H. Loosen, Joao Gorgulho, Markus S. Jördens, Maximilian Schulze-Hagen, Fabian Beier, Mihael Vucur, Anne T. Schneider, Christiane Koppe, Alexander Mertens, Jakob N. Kather, Frank Tacke, Verena Keitel, Tim H. Brümmendorf, Christoph Roderburg, Tom Luedde

**Affiliations:** ^1^ Clinic for Gastroenterology, Hepatology and Infectious Diseases, University Hospital Düsseldorf, Medical Faculty of Heinrich Heine University Düsseldorf, Düsseldorf, Germany; ^2^ Department of Medicine III, University Hospital RWTH Aachen, Aachen, Germany; ^3^ Division of Gastroenterology, Hepatology and Hepatobiliary Oncology, University Hospital RWTH Aachen, Aachen, Germany; ^4^ Department of Oncology, Hematology and Bone Marrow Transplantation with Section of Pneumology, University Medical Centre Hamburg-Eppendorf, Hamburg, Germany; ^5^ Department of Diagnostic and Interventional Radiology, University Hospital RWTH Aachen, Aachen, Germany; ^6^ Department of Medicine IV, University Hospital RWTH Aachen, Aachen, Germany; ^7^ Department of Hepatology and Gastroenterology, Charité University Medicine Berlin, Berlin, Germany

**Keywords:** immunotherapy, checkpoint inhibitors, prognosis, biomarker, nivolumab, pembrolizumab, IRAE

## Abstract

**Background:**

Immune checkpoint inhibitors (ICIs) have led to a paradigm shift in cancer therapy, improving outcomes in the treatment of various malignancies. However, not all patients benefit to the same extend from ICI. Reliable tools to predict treatment response and outcome are missing. Soluble urokinase plasminogen activator receptor (suPAR) is a marker of immune activation, whose levels are prognostic in various cancers. We evaluated circulating suPAR levels as a novel predictive and prognostic biomarker in patients receiving ICI therapy for solid tumors.

**Methods:**

A total of n = 87 patients receiving ICI therapy for different solid malignancies as well as 32 healthy controls were included into this study. Serum levels of suPAR were measured by ELISA prior to and sequentially at two time points during ICI therapy.

**Results:**

Baseline suPAR serum levels were significantly higher in solid tumor patients compared to healthy controls. Importantly, patients with low suPAR levels both before or during ICI treatment were more likely to have a favorable response to treatment at three and six months, respectively. This finding was confirmed by multivariate binary logistic regression analysis including several clinicopathological parameters. Moreover, circulating suPAR levels before and during therapy were an independent prognostic factor for overall survival (OS). As such, patients with initial suPAR levels above our ideal prognostic cut-off value (4.86 ng/ml) had a median OS of only 160 days compared to 705 days for patients with suPAR levels below this cut-off value. Finally, low baseline suPAR levels identified a subgroup of patients who experienced ICI-related side effects which in turn were associated with favorable treatment response and outcome.

**Conclusion:**

Our data suggest that measurements of suPAR serum levels are a previously unknown, easily accessible tool to predict individual treatment response and outcome to ICI therapy. Circulating suPAR might therefore be implemented into stratification algorithms to identify the ideal candidates for ICI treatment.

## Background

Cancer is the second leading cause of death in highly developed areas of the world such as Europe and the US (1). In 2013, cancer immunotherapy, or more precisely immune checkpoint inhibitors (ICIs), was deemed the “breakthrough of the year” by Science magazine (2). Several ICIs, mostly targeting the programmed cell death (PD)-L1/PD-1 (e.g. nivolumab, pembrolizumab) or the B7/cytotoxic T-lymphocyte-associated protein (CTLA)-4 (e.g. ipilimumab) pathway, have been approved either alone or in combination with e.g. chemotherapy for treatment of various cancer entities such as non-small cell lung cancer (NSCLC), malignant melanoma or urothelial carcinoma (3–5). However, tumor response rates and outcome to ICIs are very heterogeneous. While ICIs can achieve higher response and survival rates compared to conventional chemotherapy in several tumor entities, a subset of patients does not respond to immunotherapy. Although several, mostly tissue based, markers such as the expression of PD-L1, the tumor mutational burden (TMB) or the microsatellite instability (MSI) status have been suggested to predict ICI treatment response in selected tumor entities (3, 6–8), the identification of the ideal ICI patients who particularly benefit from ICI has remained challenging.

The soluble urokinase plasminogen activator receptor (suPAR) represents the cleavage product from the membrane-bound form of urokinase plasminogen activator receptor (uPAR/CD87) that is e.g. expressed on epithelial and immune cells (9). Circulating suPAR has recently been associated with inflammatory diseases and several cancer entities (10–15). During systemic inflammation, an increased shedding of uPAR on circulating neutrophils has been reported as a source of elevated suPAR levels (16). However, currently no data on a potential role of uPAR/suPAR in the context of ICIs exist. In the present study, we therefore aimed at evaluating a potential predictive and/or prognostic role of circulating suPAR as a novel biomarker in patients receiving ICIs for different solid tumor entities at the interdisciplinary tumor outpatient clinic of the University Hospital RWTH Aachen between 2018 and 2020.

## Methods

### Study Design and Patient Characteristics

This observational cohort study was designed to evaluate a potential predictive and prognostic role of circulating suPAR in a cohort of patients receiving immune checkpoint inhibitors (ICI) for different tumor entities. A total of n = 87 patients who received ICI at the interdisciplinary cancer outpatient clinic at University Hospital RWTH Aachen for advanced stage disease were prospectively recruited 2018 and 2020 and enrolled into this study (see **Table 1**). Patient characteristics such as the Eastern Cooperative Oncology Group (ECOG) performance status were assessed by a trained physician during study enrollment based on established classification systems (17). Blood samples were drawn prior to ICI therapy as well as during the course of treatment (early time point: after one or two cycles of ICI, late time point: after three, four, or five cycles of ICI). Samples were then centrifuged for 10 min at 2,000*g*, and serum samples were stored in the RWTH centralized Biomaterial Bank at −80°C until use. As a control population we analyzed a total of n = 32 healthy, cancer-free blood donors with normal values for blood counts, C-reactive protein, kidney and liver function. The study protocol was approved by the ethics committee of the University Hospital RWTH Aachen, Germany (EK 206/09) and conducted in accordance with the ethical standards laid down in the Declaration of Helsinki. Written informed consent was obtained from the patients.

**Table 1:** Patient characteristics.

Parameter	Study cohort	Baseline suPAR levels [ng/ml, median and IQR]
Cancer patients	n = 87	5.36 (2.81)
Gender [%]: male female	67.8 (n = 59) 32.2 (n = 28)	5.36 (3.24) 5.40 (3.05)
Age [years, median and range]	67.0 [38.0–87.0]	
BMI [kg/m^2^, median and range]	24.1 [15.9–42.3]	
Tumor localization [%]: NSCLC Malignant melanoma Urothelial cancer GI cancer Head and neck cancer Others	36.8 (n = 32) 14.9 (n = 13) 13.8 (n = 12) 14.9 (n = 13) 10.3 (n = 9) 9.2 (n = 8)	5.32 (3.40) 3.91 (2.34) 5.69 (2.30) 6.25 (2.63) 6.70 (2.27) 4.83 (2.51)
Staging [%]: UICC III UICC IV	6.0 (n = 5) 94.0 (n = 79)	
ICI regimen [%]: Nivolumab Pembrolizumab Nivolumab + Ipilimumab Others (e.g. Avelumab, Durvalumab)	57.5 (n = 50) 25.3 (n = 22) 9.2 (n = 8) 8.0 (n = 7)	5.53 (2.97) 6.01 (3.21) 4.86 (3.02) 4.00 (2.06)
Previous systemic therapy before ICI? [%]: Yes No	70.1 (n = 61) 29.9 (n = 26)	5.62 (2.70) 4.80 (2.47)
ECOG PS [%]: ECOG 0 ECOG 1 ECOG 2	7.1 (n = 6) 52.9 (n = 45) 40.0 (n = 34)	5.14 (3.01) 4.98 (3.47) 5.64 (2.29)
Smoking status [%]: Never Previous Present unknown	8.0 (n = 7) 42.2 (n = 35) 19.5 (n = 17) 32.2 (n = 28)	4.95 (3.23) 5.70 (3.24) 5.57 (2.25) 4.92 (2.98)
Disease control at 3 months? [%]: Yes No	47.1 (n = 41) 52.9 (n = 46)	4.68 (3.04) 5.78 (2.19)
Disease control at 6 months? [%]: Yes No	39.1 (n = 34) 60.9 (n = 53)	4.66 (3.37) 5.65 (2.29)
Disease control at 12 months? [%]: Yes No	29.6 (n = 24) 70.4 (n = 57)	4.74 (2.99) 5.65 (2.39)
Deceased during follow-up? [%]: Yes No	62.1 (n = 54) 37.9 (n = 33)	5.68 (2.21) 4.63 (3.02)
Side effects to ICI? [%]: Yes No	42.5 (n = 37) 57.5 (n = 50)	4.75 (2.44) 5.99 (2.86)
Healthy controls	n = 32	1.55 (0.635)

BMI, body mass index; NSCLC, non-small cell lung cancer; GI, gastrointestinal; UICC, Union for International Cancer Control; ICI, immune checkpoint inhibitor; ECOG PS, Eastern Cooperative Oncology Group performance status.

### Assessment of Tumor Response, Overall Survival and Immune Related Adverse Events (IRAE)

Tumor response to ICI therapy was assessed on cross-sectional imaging modalities (CT or MRI scan) at three, six and twelve months based using the RECIST v1.1 criteria where applicable (18). Tumor response was classified using the standard nomenclature for RECIST: Complete response (CR), partial response (PR), stable disease (SD) and progressive disease (PD). CR, PR and SD were defined as “disease control” (DC) whereas patient with PD were classified into non-DC. Patients who died during the respective follow-up period were defined as non-DC. Patients were followed-up by a doctor with a specialization in oncology before every administration of ICI depending on the therapy regimen as well as in between therapy cycles. Overall survival (OS) was defined as time from the first administration of ICI to death. The median follow-up time of the study cohort (first ICI therapy to death/”last follow-up”) was 261 days (IQR: 418). Immune related adverse events (IRAE) were assessed during follow up by a doctor with a specialization in oncology according to the Common Terminology Criteria for Adverse Events (CTCAE) classification. The following IRAE were documented during follow up (number of patients): hypothyroidism (four), hyperthyroidism (two), hepatitis (four), gastritis (two), pruritus/rash (nine), pneumonitis (six), colitis (four), rheumatic (two), myositis (two), vitiligo (one), pancreatitis (one).

### Measurements of suPAR Serum Levels and Routine Laboratory Parameters

Serum levels of suPAR were measured using a commercial enzyme-linked immunosorbent assay (ELISA) according to the manufacturer’s instructions (Nr. A001, suPARnostic, ViroGates, Birkerød, Denmark). Routine laboratory markers were analyzed in the central laboratory at University Hospital RWTH Aachen using a Sysmex XN9000 (Sysmex GmbH, Norderstedt, Germany) and Cobas 8000 c701 (Hoffmann-La Roche AG, Basel, Switzerland) platform according to manufacturer’s instructions.

### Statistical Analysis

Shapiro–Wilk-Test was used to test for normal distribution. Non-parametric data were compared using Mann–Whitney-U-Test and Kruskal–Wallis-Test. Related samples were compared using Wilcoxon signed-rank test. Box plot graphics display the median, quartiles and ranges. We generated receiver operating characteristics (ROC) curves by plotting the sensitivity against 1-specificity. Optimal cut-off values for ROC curves were calculated with the Youden-Index (YI) method (YI = sensitivity + specificity − 1). The predictive value of variables on treatment response was evaluated by uni- and multivariate binary logistic regression analyses. Parameters with a p-value of <0.250 in univariate testing were included into multivariate testing. The Odds ratio (OR) and 95% confidence interval are shown. Kaplan–Meier curves display the impact of a specific parameter on the overall survival (OS). The Log-rank test was used to test for statistical differences between subgroups. The ideal cut-off value for the identification of patients with an impaired OS was calculated by fitting Cox proportional hazard models to the dichotomized survival status as well as the survival time and defining the optimal cut-off as the point with the most significant split in the log-rank test. The prognostic value of variables was further tested by uni- and multivariate Cox regression analyses. Parameters with a p-value of <0.250 in univariate testing were included into multivariate testing. The hazard ratio (HR) and 95% confidence interval are displayed. In survival analyses of longitudinal suPAR alterations between baseline and the “early/late time-point”, patients who died before this time-point were excluded from analysis. All statistical analyses were performed with SPSS 23 (SPSS, Chicago, IL, USA) and RStudio 1.2.5033 (RStudio Inc., Boston, MA, USA) (11). A p-value of <0.05 was considered statistically significant (*p <0.05; **p <0.01; ***p <0.001).

## Results

### Patient Characteristics and Baseline suPAR Serum Levels

A total of n = 87 patients with advanced tumor stage scheduled to receive immune checkpoint inhibitor (ICI) therapy were included into this study prior to the first ICI administration. The median patients’ age was 67 years (range: 38–87 years). 32.2% of patients were female and 67.8% were male. NSCLC represented the most common disease etiology (36.8%), followed by malignant melanoma (14.9%), urothelial carcinoma (13.8%), GI cancer (14.9%), head and neck cancer (10.3%) and others (9.2%). Most patients (94.0%) presented with metastasized tumor stage (Union for International Cancer Control (UICC) IV), while 6.0% had UICC III tumor stage. 47.1% (n = 41/87), 39.1% (n = 34/87) and 29.6% (n = 24/81) of patients showed disease control at three, six and 12 months, respectively. **Table 1** provides a detailed overview of the study population.

**Figure 1:**
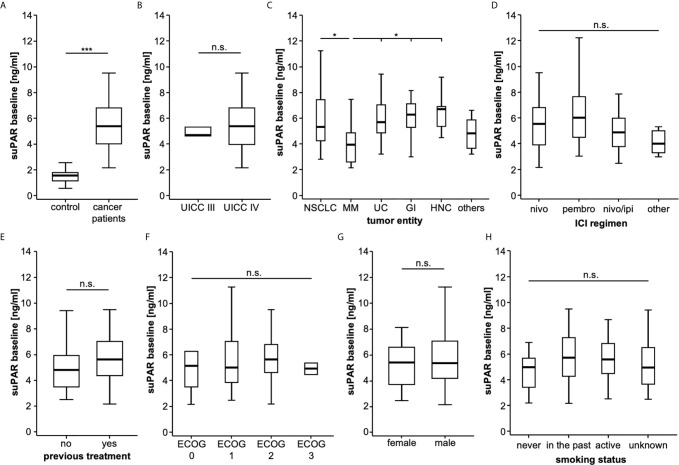
SuPAR serum levels are significantly increased in cancer patients. **(A)** SuPAR serum levels are significantly elevated in cancer patients compared to healthy controls. While baseline suPAR levels are comparable between patients with different tumor stage **(B)**, patients with malignant melanoma have significantly lower suPAR levels compared to most other tumor entities **(C)**. There is no significant difference in baseline suPAR levels regarding the scheduled ICI regimen **(D)** as well as between patients who did or did not receive previous systemic cancer therapy **(E)**. There is no regulation of circulating suPAR with respect to the ECOG performance status **(F)**, gender **(G)** or the smoking status **(H)** n.s. non significant, *p < 0.05, ***p < 0.001.

To gain first insight into the regulation of circulating suPAR in patients with advanced stage cancer, we first compared serum suPAR levels in solid tumor patients and healthy controls without signs of malignant disease. Here, we observed 3.5-fold higher suPAR serum levels in cancer patients (median: 5.36 ng/ml) compared to healthy controls (median: 1.55, **Figure 1A**). While baseline suPAR levels were comparable between patients with different tumor stage (UICC stage III vs. IV, **Figure 1B**), we observed significantly lower levels in patients with malignant melanoma (MM) compared to most other tumor entities (**Figure 1C**). There was no significant difference in baseline suPAR levels regarding the scheduled ICI regimen (**Figure 1D**) as well as between patients who did or did not receive systemic cancer therapy previously (**Figure 1E**). We did also not observe a significant regulation of circulating suPAR with respect to different patient characteristics such the ECOG performance status, sex or the smoking status (**Figures 1F–H**). However, we observed a significant positive correlation between circulating suPAR levels and the neutrophil-to-lymphocyte ratio (NRL, r_S_: 0.306, p = 0.005), which was shown to be a predictor of treatment response to ICI therapy (19).

### Baseline suPAR Serum Levels Predict Treatment Response to Immune Checkpoint Inhibitors

**Figure 2:**
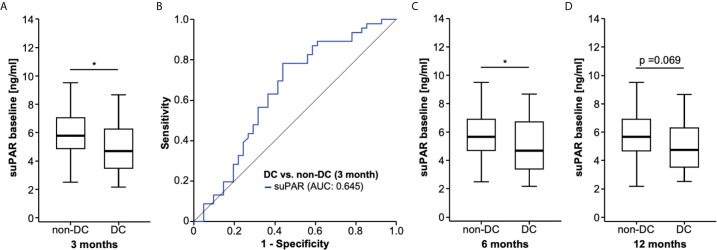
Initial suPAR serum levels predict treatment response to ICI. **(A)** Patient who show disease control (DC) to ICI at 3 months have significantly lower baseline suPAR concentrations compared to non-DC patients. **(B)** ROC curve analysis reveals an AUC value of 0.645 for suPAR regarding the discrimination between DC and non-DC patients. Initial suPAR levels are significantly lower in patients with DC at 6 months **(C)** and show a strong trend (p = 0.069), **(D)** towards lower suPAR levels at 12 months compared to non-DC patients at the respective time points. *p<0.05.

Given its important role in the context of immune activation, we next hypothesized that suPAR serum levels before the first administration of ICI could have a predictive role regarding tumor response to immunotherapy. We therefore compared baseline suPAR levels between patients who showed “disease control” (DC, n = 41, see *Methods* for detail) and patient with progressive disease (non-DC, n = 46) in the first staging scan at approximately three months after ICI therapy initialization. Strikingly, non-DC patients (median suPAR: 5.78 ng/ml) had significantly higher baseline suPAR concentrations compared to DC patients (median: 4.68 ng/ml, **Figure 2A**). ROC curve analysis revealed an AUC value of 0.645 for suPAR regarding the discrimination between DC and non-DC patients (**Figure 2B**). At the ideal predictive cut-off value of 4.804 ng/ml, suPAR showed a sensitivity and specificity of 78.3 and 56.1%. The discriminatory value of circulating suPAR was further confirmed by uni- and multivariate binary logistic regression analysis including several clinicopathological parameters (age, sex, UICC tumor stage and ECOG performance status) as well as standard laboratory markers of organ dysfunction (e.g., leucocyte count, bilirubin, creatinine, electrolytes, **Table 2**). Importantly, multivariate analysis revealed a pre-ICI suPAR concentration above 4.804 ng/ml as an independent predictor of non-DC at three months (OR: 0.215 [95% CI: 0.081–0.573], p = 0.002, **Table 2**). **Supplementary Table 1** provides a descriptive overview of baseline suPAR levels between DC/non-DC patients stratified by tumor entity and ICI regimen.

**Table 2:** Uni- and multivariate binary logistic regression analysis for the prediction of tumor response to checkpoint inhibitors.

	univariate binary logistic regression		multivariate binary logistic regression	
Parameter	p-value	Odds-Ratio (95% CI)	p-value	Odds-Ratio (95% CI)
suPAR pre-ICI >4.804 ng/ml	0.001	0.217 (0.085–0.553)	0.002	0.215 (0.081–0.573)
Age	0.515	0.987 (0.948–1.027)		
Sex	0.822	1.104 (0.466–2.617)		
UICC tumor stage	0.200	0.330 (0.061–1.800)	0.602	0.573 (0.070–4.663)
ECOG PS	0.490	0.793 (0.411–1.531)		
Leukocyte count	0.591	0.994 (0.971–1.017)		
Sodium	0.285	1.065 (0.949–1.196)		
Potassium	0.056	2.242 (0.979–5.133)	0.040	2.528 (1.043–6.125)
AST	0.321	0.992 (0.976–1.008)		
Bilirubin	0.251	0.483 (0.139–1.676)		
Creatinine	0.357	1.388 (0.691–2.788)		
LDH	0.285	1.002 (0.998–1.006)		

suPAR, soluble urokinase plasminogen activator receptor; AST, aspartate transaminase; ECOG PS, “Eastern Cooperative Oncology Group” performance status.

In a next step, we evaluated if initial suPAR levels might also be predictive for a prolonged response to ICI and compared suPAR levels in patients with DC at six or 12 months and patients who had progressed or died during this period of time (non-DC). Again, we observed significantly lower initial suPAR levels in patients with DC at six months and a non-significant trend (p = 0.069) towards lower suPAR levels in patients with DC at 12 months compared to non-DC patients at the respective time points (**Figures 2C, D**), indicating that baseline suPAR levels also predict a more durable ICI treatment response.

### Baseline suPAR Levels Predict Overall Survival in Patients Receiving Immune Checkpoint Inhibitor Therapy

**Figure 3:**
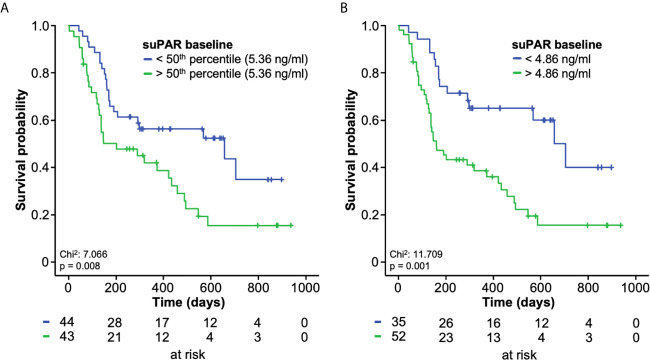
Baseline suPAR levels predict overall survival in patients receiving ICI. **(A)** Using the median suPAR concentration (5.36 ng/ml) as a cut-off, patients with initial suPAR levels above this cut-off show a significantly reduced OS compared to patients with low baseline suPAR levels. **(B)** When applying the optimal cut-off value (4.86 ng/ml), patients with a baseline suPAR concentration >4.86 ng/ml have a median OS of just 160 days compared to 705 days for patients with initial suPAR concentrations below the ideal cut-off.

We next hypothesized that baseline suPAR levels might also be indicative for the patients’ overall outcome. We therefore compared the overall survival (OS) of patients who presented with high or low circulating suPAR levels before initiation of ICI treatment. When using the median suPAR concentration (5.36 ng/ml) as a cut-off, patients with initial suPAR levels above this cut-off showed a significantly reduced OS compared to patients with low baseline suPAR levels (**Figure 3A**). The median OS was 202 days (standard error (SE): 107.4) and 658 days (293.5), respectively. We subsequently established an optimal prognostic cut-off value (see *Methods* for details). Using this optimal cut-off value of 4.86 ng/ml, initial suPAR serum levels were highly predictive for the patients’ OS. As such, patients with a baseline suPAR concentration >4.86 ng/ml had a median OS of just 160 days (SE: 36.8) compared to 705 days (SE: 88.4) for patients with initial suPAR concentrations below the ideal cut-off (**Figure 3B**). Patient characteristics of the suPAR high/low group are displayed in **Table 3**. We next performed uni- and multivariate Cox-regression analyses to identify potential confounders on patients’ outcome. In univariate analysis, baseline suPAR concentrations above the ideal cut-off value were highly predictive for OS (HR: 2.735 [95%CI: 1.501–4.985], p = 0.001, **Table 4**). Testing a broad variety of clinicopathological parameters and laboratory markers of organ dysfunction, we identified the ECOG PS, BMI, leucocyte count as well as sodium and AST levels as parameters of potential prognostic relevance for our cohort (p <0.250 in univariate analysis, **Table 4**). Importantly, in multivariate Cox-regression analysis including these parameters, baseline suPAR levels above the ideal cut-off value turned out as an independent prognostic factor for OS (HR: 2.402 [95%CI: 1.250–4.616], p = 0.009, **Table 4**).

**Table 3:** Comparison of clinical and pathological factors among patients with baseline suPAR levels below/above the ideal prognostic cut-off value.

	baseline suPAR level <4.86 ng/ml	baseline suPAR level >4.86 ng/ml
Total number of patients [n]:	35	52
Gender [n, (%)]:		
female/male	13/22 (37.1/62.9)	15/37 (28.8/71.2)
Age [years, median and range]	67 (45–87)	67 (38–87)
BMI [kg/m^2^, median and range]	25.7 (17–41.4)	22.4 (15.9–42.3)
Staging [n, (%)]:		
UICC III/UICC IV	3/32 (8.6/91.4)	2/47 (4.1/95.9)
Previous systemic therapy before ICI? [n, (%)]:		
Yes/No	15/20 (42.9/57.1)	11/41 (21.2/78.8)
ECOG PS [n, (%)]:		
ECOG 0	3 (8.8)	3 (6)
ECOG 1	19 (55.9)	26 (52)
ECOG 2	12 (35.3)	21 (42)
Smoking status [n, (%)]:		
Never	3 (8.6)	4 (7.7)
Previous	13 (37.1)	22 (42.3)
Present	6 (17.1)	11 (21.2)
unknown	13 (37.2)	15 (28.8)

BMI, body mass index; ECOG PS, “Eastern Cooperative Oncology Group” performance status; ICI, immune checkpoint inhibitor; suPAR, soluble urokinase plasminogen activator receptor; UICC, Union for International Cancer Control.

**Table 4:** Uni- and multivariate Cox-regression analysis for the prediction of overall survival.

	univariate Cox-regression		multivariate Cox-regression	
Parameter	p-value	Hazard-Ratio (95% CI)	p-value	Hazard-Ratio (95% CI)
suPAR pre-ICI >4.86 ng/ml	0.001	2.735 (1.501–4.985)	0.009	2.402 (1.250-4.616)
Age	0.927	1.001 (0.975–1.028)		
Sex	0.486	0.820 (0.468–1.435)		
BMI	0.011	0.932 (0.882–0.984)	0.102	0.953 (0.899–1.010)
UICC tumor stage	0.267	3.078 (0.423–22.386)		
ECOG PS	0.020	1.644 (1.083–2.495)	0.055	1.542 (0.991–2.401)
Leukocyte count	0.009	1.004 (1.001–1.008)	0.193	1.003 (0.998–1.008)
Sodium	0.133	0.952 (0.893–1.015)	0.872	1.006 (0.933–1.085)
Potassium	0.782	0.932 (0.564–1.539)		
AST	0.245	1.005 (0.996–1.015)	0.929	0.999 (0.988–1.012)
Bilirubin	0.864	1.074 (0.477–2.416)		
Creatinine	0.492	0.864 (0.569–1.311)		
LDH	0.644	0.999 (0.997–1.002)		

suPAR, soluble urokinase plasminogen activator receptor; AST, aspartate transaminase; BMI, body mass index; ECOG PS, “Eastern Cooperative Oncology Group” performance status; LDH, lactase dehydrogenase.

### Prognostic Relevance of Circulating suPAR During ICI Treatment

**Figure 4:**
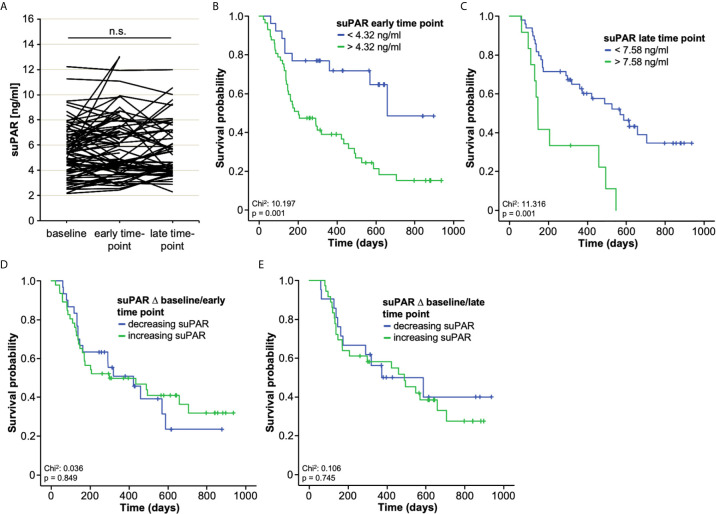
Prognostic relevance of circulating suPAR during ICI treatment. **(A)** Serum suPAR levels at the early and late time point during ICI treatment are unaltered compared to initial concentrations. **(B, C)** Patients with suPAR serum levels above the respective optimal cut-off value (early time point: 4.32 ng/ml, late time point: 7.58 ng/ml) show a significantly impaired OS compared to patients with lower suPAR levels during the course of ICI treatment. **(D, E)** There is no survival benefit in patients who show increasing or decreasing suPAR concentrations at the early or late time point compared to baseline levels. n.s., non significant.

We subsequently investigated a potential role of longitudinally assessed serum concentrations during ICI treatment at an early (after one or two cycles of immunotherapy) and a late (after three, four, or five cycles of immunotherapy) time point. Serum suPAR levels were available for a total of n = 76 and n = 57 patients at the early and late time point, respectively, and were not significantly altered compared to initial concentrations (p_early_: 0.100 and p_late_: 0.069, **Figure 4A**). In addition, suPAR serum levels at both time points did not significantly differ between patients with different tumor stage, tumor entity, ECOG PS, ICI regimen as well as male and female patients and patient with different smoking status (**Supplementary Figures 1**, **2**).

When comparing suPAR serum levels at the early and late time point during ICI treatment between patients with disease control (DC) and non-DC patients at three, six and 12 months, we observed significantly higher suPAR levels in non-DC patients at three and six months as well as a trend towards higher suPAR levels in non-DC patients at 12 months (**Supplementary Figures 3A–F**).

To analyze whether circulating suPAR levels also maintain their prognostic potential during the course of ICI therapy, we again established ideal prognostic cut-off values for the early and late time point. Similar to our previous results, patients with suPAR serum levels above the respective optimal cut-off value (early time point: 4.32 ng/ml, late time point: 7.58 ng/ml) showed a significantly impaired OS compared to patients with lower suPAR levels during the course of ICI treatment (**Figures 4B, C**). In line, univariate Cox-regression analyses confirmed the prognostic relevance of circulating suPAR above the ideal cut-off values for both the early and the late time point (HR_early_: 3.059 [95%CI: 1.486-6.297], p=0.002; HR_late_: 3.288 [95%CI: 1.578-6.852, p=0.001). Finally, we evaluated whether the longitudinal kinetic of circulating suPAR levels during ICI treatment might be indicative for the patients’ outcome. We therefore compared the OS of patients who showed increasing suPAR levels between baseline and the early time point (n=30) to those patients with decreasing suPAR concentrations (n=46). However, we did not observe a significant difference of OS between the two groups (**Figure 4D**). There was also no survival benefit in patients who showed increasing (n=21) or decreasing (n=36) suPAR concentrations at the late time point compared to baseline levels (**Figure 4E**). In line, univariate Cox-regression analysis revealed no prognostic relevance of the individual suPAR kinetic at the early or late time point (HR_baseline/early_: 0.943, 95%CI: 0.518-1.718, p= 0.849; HR_baseline/late_: 1.127, 95%CI: 0.547-2.32, p= 0.745). A comparative analysis of overall survival between patients with increasing or decreasing suPAR levels stratified by their baseline suPAR value (above/below the ideal prognostic cut-off value) confirmed this finding and revealed that baseline suPAR levels rather than the individual kinetic during the course of treatment were of prognostic relevance (**Supplementary Figures 4A**, **B**). However, patients with low baseline suPAR levels and further decreasing suPAR levels between baseline and the late time point showed a slightly superior outcome compared to patients with low baseline suPAR levels but increasing levels (**Supplementary Figure 4B**).

### Baseline suPAR Levels Correlate With Side Effects of ICI Therapy

**Figure 5:**
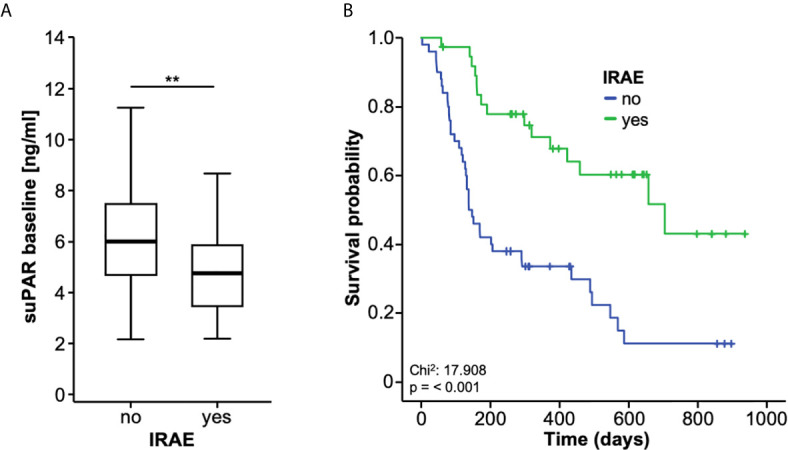
Baseline suPAR levels correlate with side effects of ICI therapy. **(A)** Patients who experience immune-related adverse events (IRAE) to ICI therapy have significantly lower baseline suPAR serum levels compared to patients without IRAE. **(B)** Overall survival is significantly higher in the subgroup of patients experiencing IRAE. **p < 0.01.

Finally, we aimed at evaluating a potential association between baseline suPAR serum levels and potential side effects to ICI during the treatment course. Here, we observed significantly lower suPAR serum levels in patients who experienced immune related adverse events (IRAE) during the course of therapy compared to patients who did not (**Figure 5A**). Interestingly, patients with IRAE showed a significantly higher rate of disease control (DC) at three months (78.4% vs. 24.0%, p <0.001), six months (64.9% vs. 20.0%, p <0.001) and 12 months (52.9 vs. 12.8%, p <0.001), respectively. Moreover, OS was significantly higher in the subgroup of patients with IRAE to ICI (**Figure 5B**).

## Discussion

Immune checkpoint inhibitors (ICI) have changed treatment paradigms for several tumor entities including NSCLC and malignant melanoma, often resulting in durable tumor responses with manageable site effects (4, 5). However, there is a subgroup of patients who do not or at least to a lesser extend benefit from ICI (20). The identification of these patients has remained challenging and up to now only very few reliable predictive markers could be established. To the best of our knowledge, we show for the first time that elevated levels of circulating suPAR predict both a poor tumor response and an impaired outcome in patients receiving ICI therapy for advanced stage solid malignancies. As such, patients with suPAR serum levels below the ideal predictive cut-off value (4.80 ng/ml) had a significantly higher probability of disease control under ICI therapy at three and six months, respectively. Moreover, baseline serum levels above the ideal prognostic cut-off value (4.86 ng/ml) were an independent prognostic factor for an impaired overall survival (OS). Patients with baseline suPAR levels above this cut-off value showed a median OS of only 160 days compared to 705 days for patients with initial suPAR concentrations below this cut-off. Finally, low baseline suPAR levels identified a subgroup of patients that experienced immune-related adverse event (IRAE) during the course of treatment, which in term was associated with a better treatment response an OS.

Although ICI are approved for an increasing number of malignancies including malignant melanoma, NSCLC and urothelial cancer (4, 5], the identification of individual patients who do not benefit from ICI therapy has remained a major challenge. Importantly, most of the existing stratification tools are tissue-based and therefore require an invasive tumor biopsy. As an example, microsatellite instability-high (MSI-H), or mismatch repair deficient (dMMR) solid tumors show a higher objective response rate to ICI, which lead to the FDA approval of pembrolizumab (anti-PD-1) as the first cancer treatment for any solid tumor with a specific genetic feature (21). Moreover, tissue expression levels of PD-L1 both in tumor and immune cells have been suggested as a predictive and/or prognostic marker in patients receiving ICI (22). In NSCLC, some ICI treatment regimens are only approved in patients with a PD-L1 tumor expression level above 50% (5). On the contrary, the potential role of PD-L1 tumor expression as a predictive marker is more controversial in other malignancies such as malignant melanoma (23). Most importantly, cut-off values for positivity (e.g. >1%, >10%, >50%), the precise cellular origin of PD-L1 expression (tumor cells, immune cells or the combination of both (combined positivity score, CPS)) and technical issues strongly vary between studies, limiting a wide-ranging clinical implementation of potential (24). In terms of circulating biomarkers, which are particularly interesting due their easy accessibility, only very few parameters have been suggested to date. In NSCLC and malignant melanoma patients receiving ICI, serum lactate dehydrogenase (LDH) levels were associated with worse survival (25, 26). A high neutrophil/lymphocyte ratio (NLR) correlated with an impaired outcome in NSCLC treated with ICI (26). In addition, serum CRP levels, a routine marker of inflammation, have been suggested to reflect treatment benefit during anti PD-(L)1 treatment in advanced NSCLC (27).

Our study provided evidence that elevated suPAR levels both before treatment initiation and during the course of treatment predict poor response and outcome to ICI. Although suPAR has been described as a prognostic marker for different treatment modalities (e.g., tumor resection, chemotherapy) of various cancer entities (10, 14, 28), this is the first study to evaluate its relevance in the context of ICI therapy. Nevertheless, the underlying molecular mechanism linking elevated suPAR levels with a poor response and outcome to ICI remains unknown. Circulating suPAR originates from shedding of the membrane bound plasminogen activator receptor (uPAR) that is expressed on immune and epithelial cells (29). During systemic inflammation, an increased shedding of uPAR on circulating immune cells, and neutrophils in particular, has been reported as a source of elevated suPAR levels (16). In proteinuric kidney disease, bone marrow-derived immature myeloid cells were identified as a main source of circulating suPAR (30). However, no data on a functional role of uPAR/suPAR in the context of ICI therapy exist to date. As a possible explanation, elevation of suPAR in the subgroup of patients with a poor response and outcome to ICI might reflect a chronically activated immune system, which in turn negatively influences the anti-tumoral effects of ICI. This hypothesis is corroborated by recent data suggesting markers of systemic inflammation such as the neutrophil-to-lymphocyte ratio (NLR) as negative predictive and/or prognostic markers for ICI therapy (31, 32). In line, it was shown that neutrophils dominate the NSCLC immune landscape being responsible for treatment failure under ICI therapy and a pro-inflammatory status was suggested to induce an “emergency granulopoiesis” leading to immature or poorly differentiated cells, which have been associated with tumor progression (26, 33). Importantly, we could show that circulating suPAR levels positively correlated with the NLR in our cohort of patients. Moreover, as it was recently shown that uPAR and suPAR can down-regulate the tumor suppressor phosphatase and tension homologue (PTEN) (34), novel data suggesting that a loss of PTEN promotes resistance to T-cell-mediated immunotherapy are of particular relevance (35, 36). However, further molecular studies are warranted to fully dissect a potential functional role of suPAR in the context of ICI therapy.

Interestingly, we observed significantly lower baseline suPAR serum levels in patients who experienced IRAE during the course of treatment compared to patients who did not. These patients had a significantly higher disease control rate and at three, six, and 12 months as well as an improved OS. There is a growing body of evidence showing that IRAE, which are believed to represent a bystander effect from activated T-cells, predict treatment response to ICI (37, 38). In this line of thinking, baseline suPAR levels could not only be useful to predict treatment response to ICI but also to identify a subgroup of patients that are more likely to experience IRAE, which in turn could trigger specific diagnostic and/or therapeutic measures to provide these patients with an optimal medical care during ICI treatment. With respect to a potential clinical implementation of circulating suPAR for the identification of the ideal candidates for ICI therapy, we suggest that suPAR should be implemented into existing or future stratification algorithms rather than being used as single biomarker. Particularly, as various more aggressive combinations of ICI as well as their combination with conventional therapies such as chemotherapy or loco-regional therapies are currently under clinical investigation (NCT04062708, NCT03572582), measurements of baseline suPAR levels might help to identify cancer patients who might not sufficiently benefit from ICI therapy alone but could be suitable candidates for a combination of therapies.

Our results are limited by some aspects. First, the study was conducted in a basket design, meaning that we included patients with different solid tumor entities and different ICI regimens. While this approach argues for a potentially both entity- and ICI-regimen-independent role of circulating suPAR, further confirmatory studies including larger patient cohorts of a certain tumor entity (e.g., NSCLC or MM) are warranted to further dissect the role of suPAR in the context of ICI among different tumor entities. Secondly, we only included patients receiving ICI but did not evaluated suPAR levels and the clinical course of patients receiving an alternative treatment such as conventional chemotherapy. Moreover, tumor samples were not available in our cohort and we were thus unable to investigate a potential association between suPAR serum levels and established tissue-based markers of ICI treatment response such as tumoral PD-L1 expression or the TMB (8, 19). In this line of thinking, a combination of suPAR with other predictive biomarkers including patient characteristics such obesity, which was associated with a better survival of cancer patients receiving ICI therapy, should be considered (39–41). Finally, we are unable to provide information on the specific molecular mechanism being responsible for a poor treatment response and outcome to ICI in the subgroup of patients with high suPAR levels. Thus, further clinical trials including larger patient cohorts as well as molecular studies in e.g. uPAR knock-out mice (42) are warranted to corroborate our findings and to further evaluate the specific function of uPAR/suPAR in the context of ICI, which we hope to have encourage with our exploratory study.

In summary, our data suggest a previously unrecognized predictive and prognostic role of circulating suPAR in the context of ICI therapy. If these findings were confirmed in larger clinical trials, suPAR might represent a valuable stratification tool and complement existing algorithms to identify the ideal candidates for ICI in future.

## Data Availability Statement

The raw data supporting the conclusions of this article will be made available by the authors, without undue reservation.

## Ethics Statement

The studies involving human participants were reviewed and approved by Ethics committee of the University Hospital RWTH Aachen, Germany (EK 206/09). The patients/participants provided their written informed consent to participate in this study.

## Author Contributions

TL and SL designed the study. JG and SL recruited patients. SL and JG performed experiments. SL, JK, and JG performed statistical analysis and generated figures and tables. MJ, MS-H, FB, MV, AS, CK, AM, VK, TB, and CR provided intellectual input. SL, JG, and TL drafted the manuscript. All authors contributed to the article and approved the submitted version.

## Funding

Work in the lab of TL was funded from the European Research Council (ERC) under the European Union’s Horizon 2020 research and innovation program through the ERC Consolidator Grant PhaseControl (Grant Agreement n° 771083). The lab of TL was further supported by the German Cancer Aid (Deutsche Krebshilfe 110043 and a Mildred-Scheel-Professorship), the German-Research-Foundation (SFB-TRR57/P06, LU 1360/3-1, CRC1380/A01, and CA 830/3-1), the Ernst-Jung-Foundation Hamburg, the IZKF (interdisciplinary centre of clinical research) Aachen, and a grant from the medical faculty of the RWTH Aachen. The suPAR ELISA kits were provided by Virogates (Denmark).

## Conflict of Interest

The authors declare that the research was conducted in the absence of any commercial or financial relationships that could be construed as a potential conflict of interest.

## Supplementary Material

Supplementary Figure 1 SuPAR serum levels at the early time point do not significantly differ between patients with different tumor stage **(A)** tumor entity **(B)** ECOG PS **(C)** ICI regimen **(D)** as well as male and female patients **(E)** and patient with different smoking status **(F)**.

Supplementary Figure 2 SuPAR serum levels at the late time point do not significantly differ between patients with different tumor stage **(A)**, tumor entity **(B)**, ECOG PS **(C)**, ICI regimen **(D)** as well as male and female patients **(E)** and patient with different smoking status **(F)**.

Supplementary Figure 3
**(A, B)** Patients with disease control (DC) at three months have significantly lower suPAR serum levels at the early and late time point during ICI treatment compared to non-DC patients. **(C, D)** Patients with disease control (DC) at six months have significantly lower suPAR serum levels at the early and late time point during ICI treatment compared to non-DC patients. **(E, F)** Patients with disease control (DC) at 12 months show a trend towards lower suPAR serum levels at the early and late time point during ICI treatment compared to non-DC patients.

Supplementary Figure 4
**(A)** Kaplan-Meier curve analysis evaluating the overall survival of patients with high/low baseline suPAR levels (ideal prognostic cut-off value) and increasing or decreasing suPAR values between treatment initialization and the early time point. **(B)** Kaplan-Meier curve analysis evaluating the overall survival of patients with high/low baseline suPAR levels (ideal prognostic cut-off value) and increasing or decreasing suPAR values between treatment initialization and the late time point.
