# Supplementary figures and images for: Serum Levels of Soluble Urokinase Plasminogen Activator Receptor Predict Tumor Response and Outcome to Immune Checkpoint Inhibitor Therapy
Source: Front Oncol. 2021 Apr 1;11:646883. doi: 10.3389/fonc.2021.646883 (PMC8047604; doi:10.3389/fonc.2021.646883)

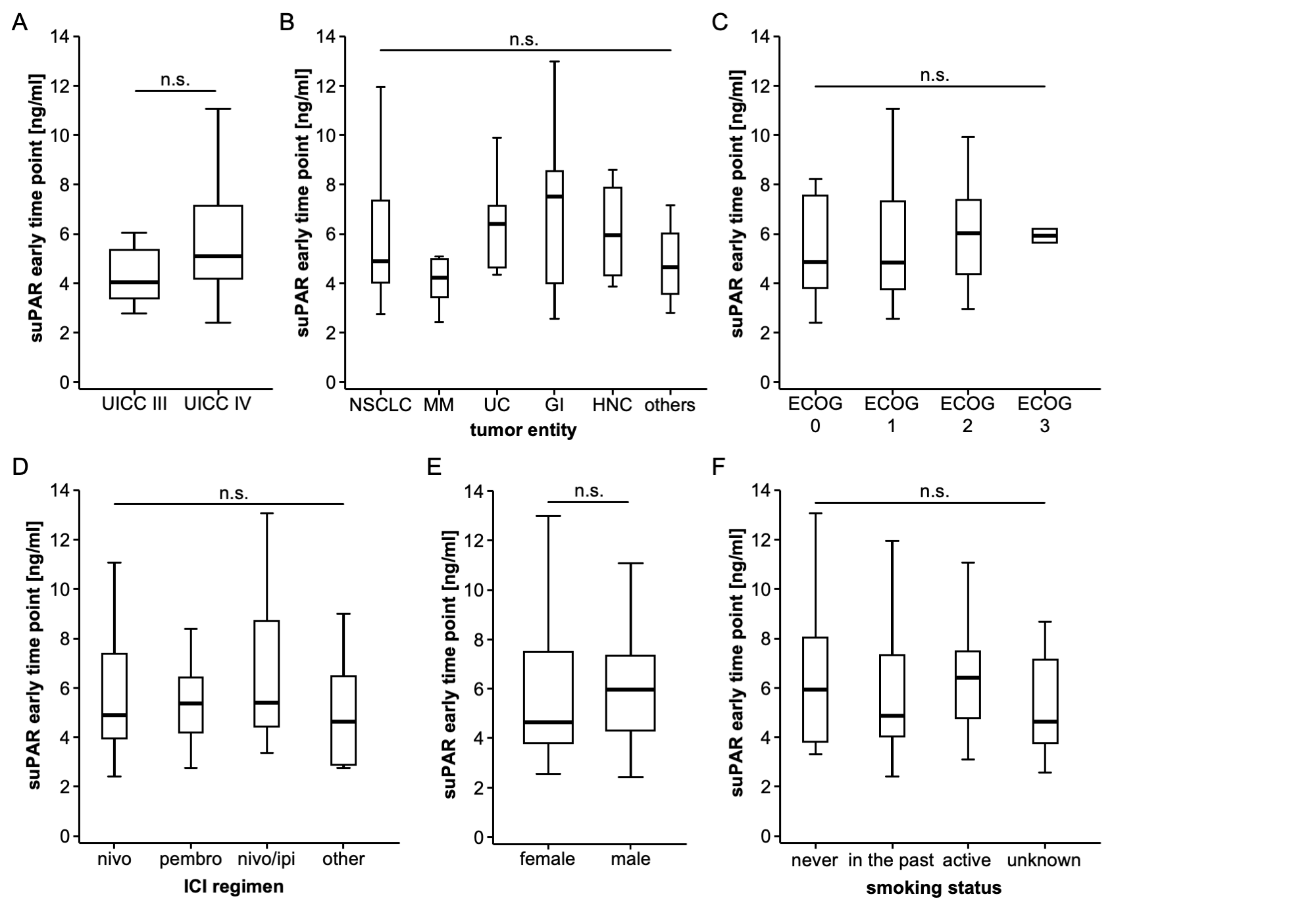

Supplement: Supplementary Figure 1 — SuPAR serum levels at the early time point do not significantly differ between patients with different tumor stage (A) tumor entity (B) ECOG PS (C) ICI regimen (D) as well as male and female patients (E) and patient with different smoking status (F). [file Image_1.tiff]

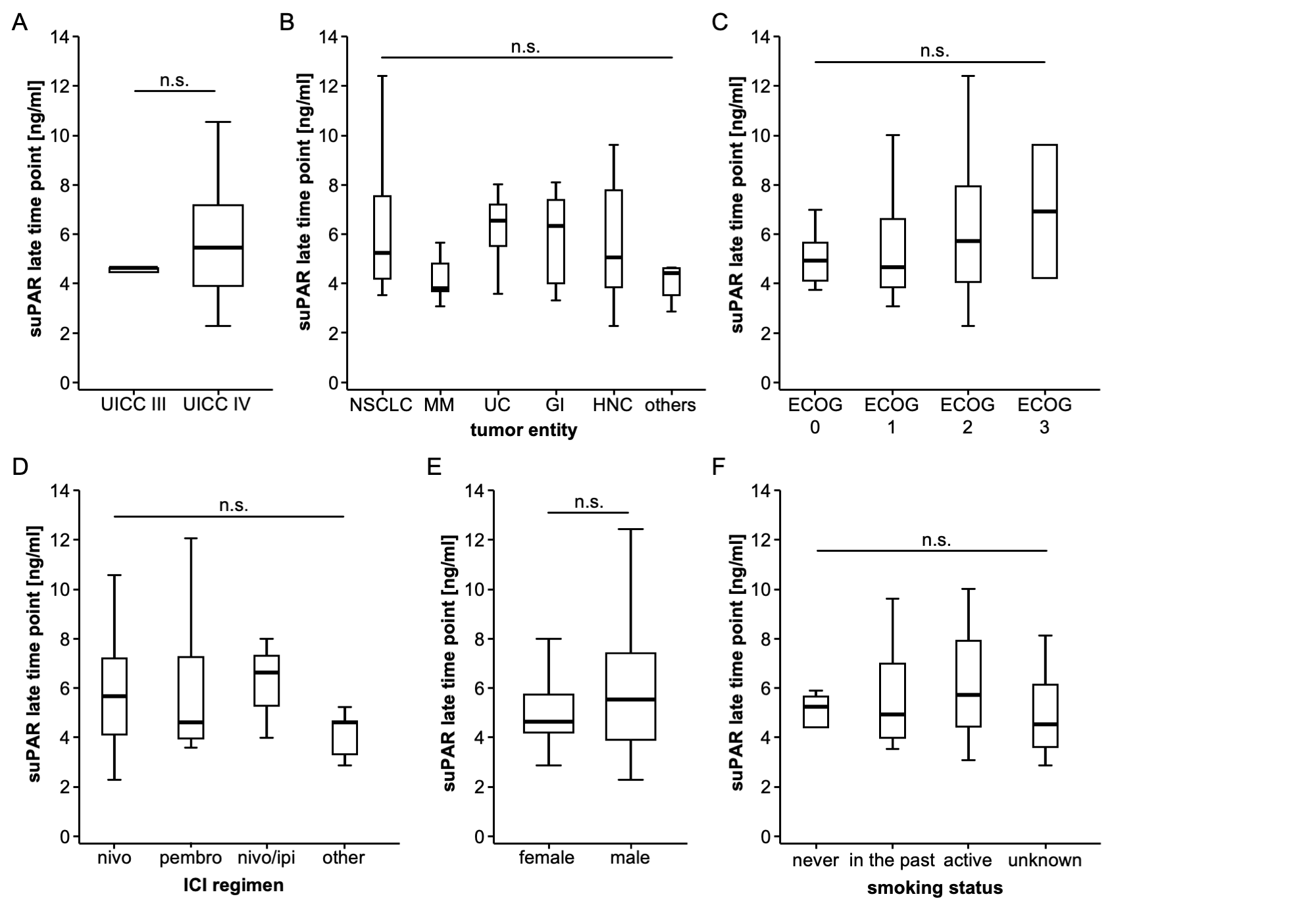

Supplement: Supplementary Figure 2 — SuPAR serum levels at the late time point do not significantly differ between patients with different tumor stage (A), tumor entity (B), ECOG PS (C), ICI regimen (D) as well as male and female patients (E) and patient with different smoking status (F). [file Image_2.tiff]

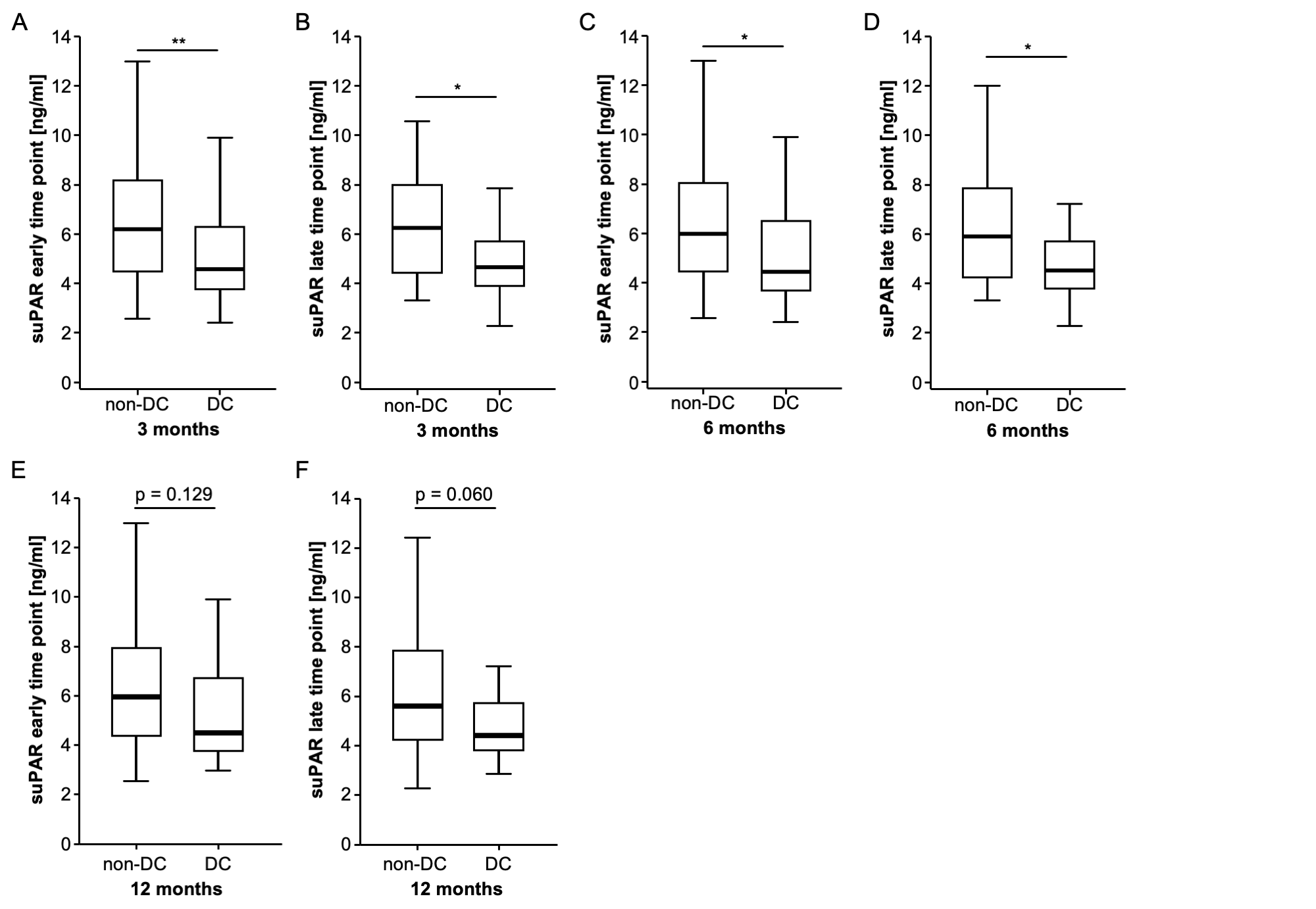

Supplement: Supplementary Figure 3 — (A, B) Patients with disease control (DC) at three months have significantly lower suPAR serum levels at the early and late time point during ICI treatment compared to non-DC patients. (C, D) Patients with disease control (DC) at six months have significantly lower suPAR serum levels at the early and late time point during ICI treatment compared to non-DC patients. (E, F) Patients with disease control (DC) at 12 months show a trend towards lower suPAR serum levels at the early and late time point during ICI treatment compared to non-DC patients. [file Image_3.tiff]

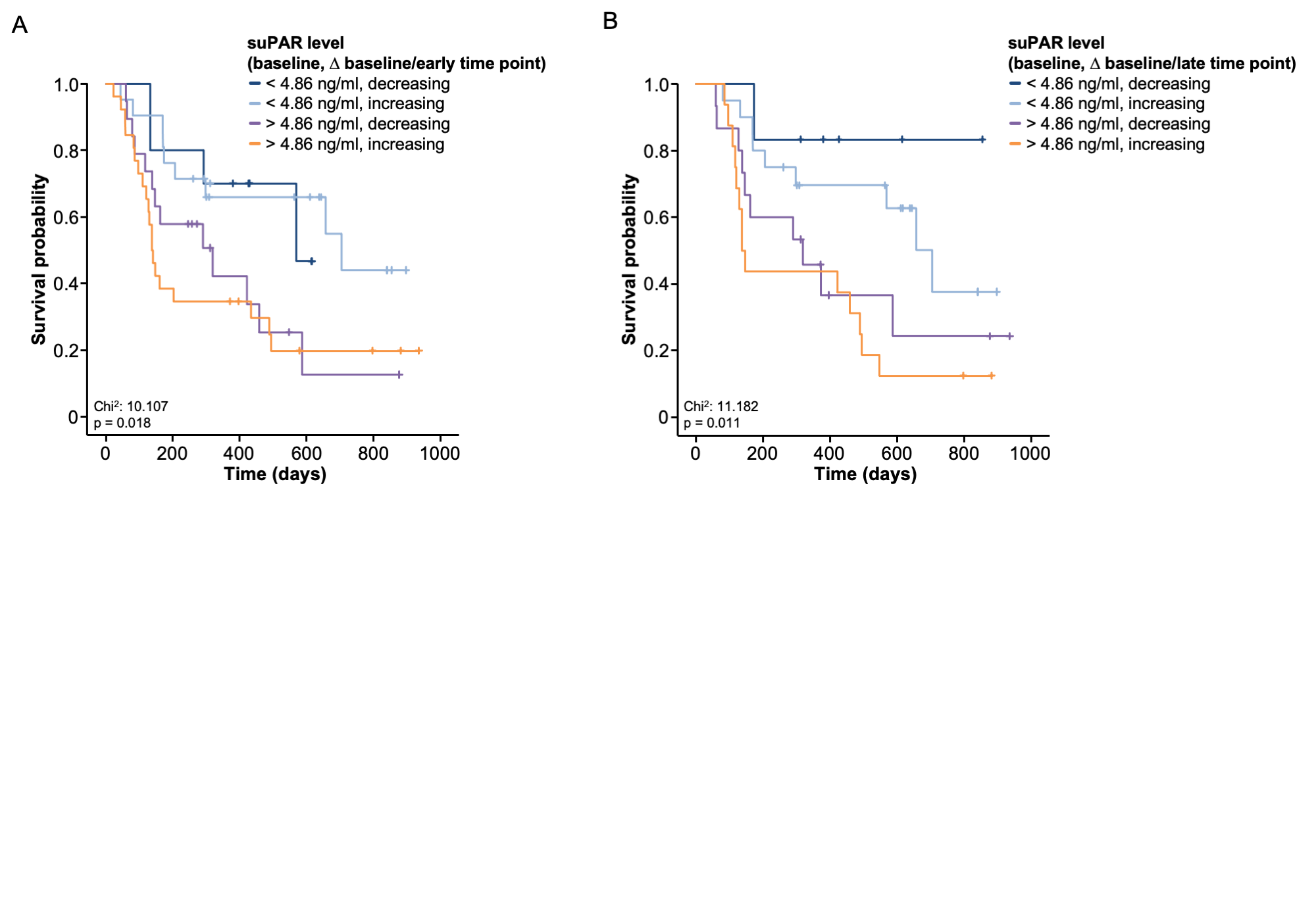

Supplement: Supplementary Figure 4 — (A) Kaplan-Meier curve analysis evaluating the overall survival of patients with high/low baseline suPAR levels (ideal prognostic cut-off value) and increasing or decreasing suPAR values between treatment initialization and the early time point. (B) Kaplan-Meier curve analysis evaluating the overall survival of patients with high/low baseline suPAR levels (ideal prognostic cut-off value) and increasing or decreasing suPAR values between treatment initialization and the late time point. [file Image_4.tiff]
