# Supplementary material for: Serum Levels of Soluble Urokinase Plasminogen Activator Receptor Predict Tumor Response and Outcome to Immune Checkpoint Inhibitor Therapy
Source: Front Oncol. 2021 Apr 1;11:646883. doi: 10.3389/fonc.2021.646883 (PMC8047604; doi:10.3389/fonc.2021.646883)
Supplement: Supplementary file 5 [file Table_1.docx]

# Supplementary Tables

**Supplementary Table 1:** Baseline suPAR levels of patients with DC or non-DC at 3 months

|  | **Baseline suPAR level** [ng/ml], median (IQR) | |
| --- | --- | --- |
| **Tumor entity** | **DC at 3 months** | **Non-DC at 3 months** |
| NSCLC | 5.03 (3.67), n=20 | 5.76 (2.95), n=12 |
| MM | 3.50 (2.43), n=11 | 4.62 (%), n=2 |
| UC | 5.69 (3.85), n=6 | 5.65 (2.58), n=6 |
| GI | % (%), n=0 | 6.25 (2.63), n=13 |
| HNC | 4.44 (%), n=2 | 6.74 (2.17), n=7 |
| others | 3.95 (%), n=2 | 5.30 (2.45), n=6 |
|  | | |
| **Treatment regimen** |  |  |
| Nivolumab | 4.37 (3.48), n=22 | 5.88 (2.00), n=28 |
| Pembrolizumab | 4.71 (3.84), n=12 | 6.57 (2.17), n=10 |
| Nivolumab + Ipilimumab | 4.07 (4.47), n=4 | 4.86 (2.00), n=4 |
| other | 4.67 (%), n=3 | 3.69 (1.89), n=4 |

DC: disease control, IQR: interquartile range, NSCLC: Non-small cell lung cancer, MM: malignant melanoma, UC: urothelial carcinoma, HNC: head and neck cancer, %: unable to calculate due to small sample size
